# On History in Acute Cases

**Published:** 1908-08-29

**Authors:** 


### Resident Medical Officers' Department.

### ON HISTORY IN ACUTE CASES.

Every medical man knows the value of a concise account of the early history in an acute case. Its value is immense both as to diagnosis, and as to treatment. And this is especially so where the case falls among those in which operative interference may be necessary. Yet many a general practitioner will send up to a hospital a case of strangulated hernia, or of general peritonitis, with simply the diagnosis written on the back of his visiting card. He forgets the difference between hospital and private practice, and the responsibilities of each. He forgets that though with him rests the responsibility of sending such a patient to the hospital, it is with the hospital surgeon that the responsibility lies whether to operate, and when to operate. The surgeon who undertakes such a responsibility must make his own diagnosis, however much confidence he may have in the practitioner. He forgets, too, that he is the one of all others who is best able to give the history of the earliest onset, and of the further progress of the illness. For when he is called in, the patient himself is still sufficiently collected to be able to give his own account of the first symptoms, and his relations have the details still in their memory, whereas by the time they arrive at the hospital, the patient, worn out with pain and suffering, and the relations, confused and upset at the hurried departure, and the thought of a possible operation, are equally unable to give anything like a concise account of the onset of the illness.

Further, during the time that elapses from his first seeing the patient and examining him, till the time when he sends him up to the hospital, the practitioner's account is of far greater importance that that of the most self-controlled relative. For he alone has been able to weigh the value of the various symptoms, and to look out for such as might miss the notice of the layman.

Such being the facts, it may not be amiss to run through a few of those points which are most valuable in the history of such cases.

### Symptoms that should be Noted.

A series of symptoms is of far more value than the most accurate diagnosis. The irreducible hernia threatening to become strangulated, may go back in the cab; the case of perforated gastric ulcer may have reached its period of quiescence by the time it arrives at the hospital. In either case if a diagnosis only be sent, it is liable to seem of little account, or to be ignored altogether, whereas an outline of the history at once impresses the seriousness of the case.

Firstly, with regard to pain, a doctor is often called in simply for pain, and this is frequently localised when he first sees the case, whereas by the time it arrives in hospital the pain has become general, and the patient has forgotten the exact original spot. If the doctor had sent a note as to the exact spot where the original pain was felt, and as to the relation of its onset to the actions of the patient, a most valuable point is gained. Biliary, renal, and appendicular pains are often distinct in their localisation at first, and diverge one into another later; pneumonia and general peritonitis may be similarly confused.

Then, again, in the important matter of the relation of vomiting to the action of the bowels: the friends know the patient has vomited, they know, too, that the bowels have not been opened, but by the time they reach the hospital they can seldom remember whether the vomiting came on before the constipation, and what is the relation of either to the onset of pain.

When a note of the temperature is sent up to the hospital with the patient, the value of a series of readings over a single one is great. Often of course this is impossible, but where possible it should be given. It sometimes happens that on arrival the temperature is some degrees below that of the single reading that is sent up. Has this fall of temperature occurred before? or does it mark some important change in the course of the disease? These are questions which it would be of inestimable benefit to know; and the same applies to pulse and respiration. Again, in acute abdominal cases a history as to rigidity of the abdomen is valuable, whether this history be positive or negative, whether a general rigidity be preceded by a localised one or not.

### A Record of Treatment.

Lastly, the treatment that the doctor has given is a valuable thing to know; often the admitting officer is faced with the story that the doctor gave the patient a bottle of medicine, and that is all. To know whether purgatives have been tried, and if so, to what extent, and in what manner, and to know the result of such treatment is invaluable, and the same may be said of enemata.

But above all, the use of opium and its preparations should be told to the hospital medical officer, or a negative history given. This cannot be given in too great a detail as to dose, to time, to manner of administration, and whether it has been repeated. To this may be added an account of the alteration of symptoms after the drug has been taken. Perhaps with regard to the giving of opium, negative evidence is of more value than it is in any other point, and it is of value both as to the fact of administration, and to its influence on the symptoms. If in every case sent up to a hospital the above points were indicated in a short note, the work of the resident officers and others would be much lightened in these important cases.

### RESIDENT STAFF APPOINTMENT.

The vacancy in the post of assistant resident medical officer to the Royal Albert Hospital, Devonport, has been filled by the appointment of Mr. H. Martin Grey, M.R.C.S., L.R.C.P.

